# Supplementary material for: Valorization of a Waste Product of Edible Flowers: Volatile Characterization of Leaves
Source: Molecules. 2022 Mar 27;27(7):2172. doi: 10.3390/molecules27072172 (PMC9000653; doi:10.3390/molecules27072172)
Supplement: Supplementary file 1 [file molecules-27-02172-s001.zip › molecules-1629637-supplementary.pdf]

Supplementary

Table S1. Volatile chemical composition of the leaves from the studied Lamiaceae species, analysed by headspace solid phase microextraction (HS-SPME) (n = 3±SD).

				**Mentheae Tribe**									**Ocimeae Tribe**		
				**Subtribe Nepetinae**					**Subtribe Menthinae**	**Subtribe Salviinae**			***Subtribe Ociminae***		
				***A.* 'Arcado Pink'**	***A. aurantiaca***	***A.*'Blue Boa'**	***A. mexicana***	***N. x faasenii***	***M. didyma***	***S. discolor***	***S. dorisiana***	***S. microphylla***	***O. b* 'Cinnamon'**	***O. × citriodorum***	***O. b* 'Blue Spice'**
**N°**	**Compounds[1]**	**class**	**LRI[2]**	*Relative abundance (%)*											
**1**	β-Myrcene	mh	991	-	-	0.1±0.03	2.6±0.13	0.1±0.01	-	-	0.5±0.02	-	-	0.3±0.13	-
**2**	Limonene	mh	1029	0.5±0.08	0.7±0.09	4.0±0.50	0.4±0.04	0.9±0.09	1.2±0.18	-	7.0±0.47	-	-	-	-
**3**	γ-Terpinene	mh	1029	-	-	-	0.1±0.06	0.3±0.09	-	-	0.1±0.01	4.6±0.10	-	-	-
**4**	Eucalyptol	om	1031	-	-	-	-	7.4±0.16	-	-	-	11.1±0.93	0.4±0.26	-	-
**5**	*(Z)*-β-Ocimene	mh	1037	-	-	-	0.4±0.03	-	-	-	-	-	-	-	2.2±0.19
**6**	*(E)*-β-Ocimene	mh	1050	-	-	-	1.0±0.48	-	-	-	-	-	2.0±0.13	-	0.4±0.09
**7**	Linalool	om	1097	-	-	-	1.5±0.46	0.1±0.01	4.5±1.37	-	-	-	2.9±0.48	-	-
**8**	Camphor	om	1146	-	-	-	0.1±0.09	-	0.2±0.02	-	-	8.4±0.20	0.4±0.03	-	-
**9**	*iso*Menthone	om	1164	2.9±0.94	-	4.7±0.11	-	-	-	-	-	-	-	-	-
**10**	Menthofurane	om	1164	-	1.0±0.26	-	-	-	-	-	-	-	-	-	-
**11**	4-Terpineol	om	1177	-	-	-	-	0.2±0.06	0.2±0.04	-	-	-	1.5±0.32	-	-
**12**	*iso*Pulegone	om	1177	-	1.8±0.15	0.6±0.07	0.1±0.01	-	-	-	-	-	-	-	-
**13**	Citronellol	om	1226	-	-	-	5.3±1.27	-	-	-	-	-	-	-	-
**14**	Nerol	om	1230	-	-	-	-	-	-	-	-	-	-	4.2±0.54	-
**15**	Thymol methyl ether	om	1235	-	-	-	-	-	13.3±3.37	-	-	-	-	-	-
**16**	Pulegone	om	1237	3.1±0.38	52.8±4.37	51.9±2.27	-	-	-	-	-	-	-	-	-
**17**	Neral	om	1238	-	-	-	2.0±0.15	-	-	-	-	-	-	**8.6±1.86**	
**18**	Chavicol	pp	1250	-	-	-	-	-	-	-	-	-	-	-	2.0±0.11
**19**	Beraniol	om	1253	-	-	-	7.1±0.29	-	-	-	-	-	-	-	-
**20**	3,7-dimethyl-2,6-octadien-1-ol	om	1253	-	-	-	-	-	-	-	-	-	-	3.3±0.91	-
**21**	Geranial	om	1267	-	-	-	5.3±0.80	-	-	-	-	-	-	**23.7±3.61**	-
**22**	*iso*Bornyl acetate	om	1286	-	-	-	1.4±0.39	-	-	-	-	-	-	-	-

**23**	Bornyl acetate	om	1289	-	-	-	-	-	1.1±0.36	-	-	0.3±0.04	-	-	-
**24**	Thymol	om	1290	-	-	-	-	0.1±0.02	37.2±1.76	-	-	-	-	-	-
**25**	Myrtenyl acetate	om	1327	-	-	-	-	-	-	-	3.4±0.17	-	-	-	-
**26**	Elemene isomer	sh	1344	2.4±0.11	-	2.6±0.92	-	-	-	-	-	-	-	-	-
**27**	Citronellyl acetate	om	1353	-	-	-	9.2±0.17	-	-	-	-	-	-	-	-
**28**	Eugenol	pp	1359	-	-	-	-	-	-	-	-	-	**28.8±2.77**	2.1±0.28	**35.6±5.67**
**29**	Nerol acetate	om	1362	-	-	-	4.2±0.05	-	-	-	-	-	-	0.5±0.04	-
**30**	α-Copaene	sh	1377	0.4±0.08	-	-	-	-	0.5±0.06	-	-	12.0±0.45	0.3±0.04	-	-
**31**	Geranyl acetate	om	1381	-	-	-	46.7±2,81	-	-	-	-	-	-	-	-
**32**	β-Bourbonene	sh	1388	1.0±0.11	-	-	-	-	0.2±0.16	0.4±0.03	-	-	-	-	-
**33**	β-Elemene	sh	1391	3.5±0.12	-	-	-	0.3±0.14	1.4±0.38	13.4±0.28	-	-	11.9±1.98	0.3±0.04	-
**34**	*cis-trans*-Nepetalactone	om	1392	-	-	-	-	37.1±2.76	-	-	-	-	-	-	-
**35**	Methyl perillate	om	1394	-	-	-	-	-	-	-	25.2±2.14	-	-	-	-
**36**	Methyleugenol	pp	1402	-	-	-	2.9±0.26	-	-	-	-	-	-	-	-
**37**	β-Caryophyllene	sh	1419	21.6±0.49	35.4±5.29	19.0±1.03	7.5±0.77	34.8±3.33	9.8±0.78	53.9±3.73	23.9±2.75	27.6±0.22	-	10.3±0.34	-
**38**	β-Ylangene	sh	1421	-	-	-	-	-	-	-	-	-	1.3±0.29	-	-
**39**	β-Copaene	sh	1432	2.9±0.08	-	-	-	-	-	-	-	-	-	-	-
**40**	*cis*-β-Copaene	sh	1432	-	0.1±0.03	-	-	0.3±0.04	1.3±0.20	-	0.1±0.01	1.0±0.07	0.5±0.19	-	-
**41**	*trans*-α-Bergamotene	sh	1435	-	-	-	-	-	-	-	-	-	0.8±0.13	5.2±0.46	1.4±0.69
**42**	Perillyl acetate	om	1436	-	-	-	-	-	-	-	23.6±1.08	-	-	-	-
**43**	α-Guaiene	sh	1440	-	-	-	-	-	-	-	-	0.1±0.07	2.6±0.02	-	-
**44**	Aromadendrene	sh	1441	-	-	-	-	-	-	-	1.2±0.19	-	-	-	-
**45**	*iso*Germacrene D	sh	1448	1.1±0.37	-	-	-	-	-	-	-	-	-	-	-
**46**	*cis*-Muurola-3,5-diene	sh	1450	-	-	-	-	-	-	-	-	-	1.1±0.38	-	-
**47**	α-Humulene	sh	1454	4.7±0.52	4.5±0.25	0.9±0.13	1.0±0.03	1.6±0.40	-	6.7±0.01	0.7±0.20	1.4±0.04	1.3±0.09	3.0±0.08	1.3±0.36
**48**	*(E)*-β-Farnesene	sh	1457	-	0.5±0.05	-		0.6±0.29	-	0.6±0.19	0.2±0.03	-	0.2±0.13	-	1.0±0.35
**49**	*cis*-Muurola-4(15),5-diene	sh	1462	2.0±0.13	-	-	-	-	0.7±0.11	-	-	-	2.0±0.35	-	-
**50**	γ-Muurolene	sh	1477	1.0±0.12	-	-	-	-	-	-	-	3.1±0.02	0.2±0.12	-	-
**51**	γ-Selinene	sh	1478	-	-	-	-	-	-	2.6±0.03	-	-		-	-
**52**	Germacrene D	sh	1481	33.3±3.04	2.1±0.78	4.1±0.22	-	6.0±0.37	23.0±3.28	2.1±0.11	0.4±0.12	-	9.1±0.45	5.2±1.04	1.6±0.46

**53**	β-Eudesmene	sh	1486	-	-	-	-	-	-	4.9±0.25	-	-	-	-	-
**54**	β-Selinene	sh	1490	-	-	-	-	-	-	-	-	2.4±0.02	0.4±0.11	-	-
**55**	ledene	sh	1493	-	-	-	-	-	-	-	1.5±0.03	-	-	-	-
**56**	Valencene	sh	1496	-	-	-	-	-	-	-	-	1.6±0.04	-	-	-
**57**	bicyclogermacrene	sh	1500	7.2±0.71	-	7.8±0.75	-	-	-	-	-	-	-	-	-
**58**	β-Bisabolene	sh	1506	-	-	-	-	0.3±0.13	-	3.8±0.76	-	-	0.2±0.06	0.3±0.09	25.9±2.22
**59**	*(E,E)*-α-Farnesene	sh	1508	1.9±0.06	-	1.6±0.08	-	-	-	-	-	-	-	-	-
**60**	α-Cuprene	sh	1509	-	-	-	-	-	-	1.3±0.08	-	-	-	-	-
**61**	α-Bulnesene	sh	1510	-	-	-	-	-	-		-	-	7.6±0.25	-	-
**62**	γ-Cadinene	sh	1513	3.1±0.65	-	-	-	0.5±0.09	-	0.9±0.25	0.8±0.03	1.5±0.02	13.0±1.11	-	-
**63**	1-ethyl-3-vinyl-adamantane	nt	1514	-	-	0.4±0.17	-	-	1.0±0.54	-	-	-	-	-	-
**64**	δ-Cadinene	sh	1523	3.6±0.91	0.1±0.06	0.3±0.14	-	0.4±0.11	1.7±0.65	-	2.3±0.12	7.7±0.06	0.9±0.16	0.4±0.14	-
**65**	*(E)*-γ-Bisabolene	sh	1531	-	-	-	-	0.1±0.09	-	-	-	-	-	**29.9±0.95**	-
**66**	Naphthalene, decahydro-4a-methyl-1-methylene-7-(1-methylethylidene)-, trans-	sh	1544	-	-	-	-	-	-	1.2±0.18	-	-	-	-	-
**67**	Germacrene B	sh	1561	-	-	-	-	-	-	-	-	-	-	-	24.9±1.90
**68**	Guaiol	os	1601	-	-	-	-	-	-	-	-	5.7±0.70	-	-	-
**69**	*epi*Cubenol	os	1627	-	-	-	-	-	-	-	-	-	1.0±0.14	-	-
**70**	γ-Eudesmol	os	1632	-	-	-	-	-	-	-	-	1.1±0.17	-	-	-
**71**	T-Cadinol	os	1640	-	-	-	-	0.1±0.03	-	-	-	-	7.3±0.28	0.8±0.08	
**72**	β-Eudesmol	os	1651	-	-	-	-	-	-	-	-	1.3±0.18	-	-	-
**73**	α-Eudesmol	os	1654	-	-	-	-	-	-	-	-	2.6±0.30	-	-	-
**74**	Elemyl acetate	sh	1679	-	-	-	-	-	-	5.1±0.75	-	-	-	-	-
**75**	Pentylcurcumene	dh	1930	-	-	-	-	4.2±1.49	-	-	1.6±0.75	-	-	-	-
**76**	9-Geranyl-*p*-cymene	od	1980	-	-	-	-		-	-	1.3±0.39	-	-	-	-

	**Mentheae Tribe**									**Ocimeae Tribe**		
	**Subtribe Nepetinae**					**Subtribe Menthinae**	**Subtribe Salviinae**			***Subtribe Ociminae***		
**Chemical classes**	***A.* ‘Arcado Pink’**	***A. aurantiaca***	***A.*‘Blue Boa’**	***A. mexicana***	***N. x faasanii***	***M. didyma***	***S. discolor***	***S. dorisiana***	***S. microphylla***	***O. b* ‘Cinnamon’**	***O. × citriodorum***	***O. b* ‘Blue Spice’**
**Monoterpene Hydrocarbons (mh)**	0.5±0.08		4.7±0.19	4.8±0.68	2.2±0.23	1.2±0.18	0.5±0.24	7.9±0.49	5.1±0.16	2.0±0.13	0.3±0.13	2.6±0.28

	A. 'Arcado Pink'	A. aurantiaca	A. 'BleuBoa'	A. mexicana	N. x faasanii	M. didyma	S. discolor	S. dorisiana	S. microphylla	O. b 'Cinnamon'	O. × citriodorum	O. b. 'Blue Spice'
**Oxygenated Monoterpenes (om)**	6.3±0.68	**56.5±4.47**	**57.9±2.77**	**83.1±1.92**	**45.0±3.36**	**56.5±5.86**	-	**53.8±4.07**	19.8±0.78	5.2±0.30	40.3±3.57	-
**Sesquiterpene Hydrocarbons (sh)**	**92.2±4.41**	42.8±5.75	36.3±1.03	8.5±0.76	**46.0±5.74**	39.9±4.18	**97.8±1.40**	32.5±2.33	**60.8±0.59**	**55.3±3.96**	**54.7±0.14**	**56.6±5.01**
**Oxygenated Sesquiterpenes (os)**	0.8±0.17	-	-	0.1±0.07	0.6±0.34	-	-	0.2±0.16	11.6±1.41	8.4±0.47	0.9±0.10	-
**Diterpene Hydrocarbons (dh)**	-	-	-	-	4.2±1.49	-	-	1.6±0.75	-	-	-	-
**Oxygenated Diterpenes (od)**	-	-	-	-	-	-	-	1.3±0.39	-	-	-	-
**Phenylpropanoids (pp)**	-	-	-	2.9±0.26	0.7±0.06	-	-	-	-	28.8±2.77	2.1±0.28	38.1±6.22
**Non-Terpene derivatives (nt)**	-	-	0.6±0.04	-	0.1±0.09	1.1±0.46	0.4±0.08	0.9±0.25	-	0.2±0.09	-	-
**Total Identified**	**99.8±0.12**	**99.3±0.09**	**99.5±0.36**	**99.4±0.31**	**98.8±0.55**	**98.7±0.08**	**98.7±0.36**	**98.2±0.50**	**97.3±0.20**	**99.9±0.01**	**98.3±0.95**	**97.3±0.60**

[1]compounds present in tables with value > 1% in at least one of studied species; [2]LRI: relative retention index determined on HP-5MS capillary column.

Table S2. Chemical composition of the EOs obtained from the leaves of the studied plant species (n = 3±SD).

				**Mentheae Tribe**									**Ocimeae Tribe**		
				**Subtribe Nepetinae**					**Subtribe Menthinae**	**Subtribe Salviinae**			***Subtribe Ociminae***		
				A. **'Arcado Pink'**	***A. aurantiaca***	***A.* 'BleuBoa'**	***A. mexicana***	***N. x faasanii***	***M. didyma***	***S. discolor***	***S. dorisiana***	***S. microphylla***	***O. b* 'Cinnamon'**	***O. × citriodorum***	***O. b.* 'Blue Spice'**
**N°**	**Compounds[1]**	**Class**	**LRI[2]**	*Relative abundance (%)*											
**1**	α-Pinene	mh	939	-	-	-	-	0.2±0.02	-	-	-	0.4±0.06	-	-	-
**2**	Camphene	mh	954	-	-	-	0.3±0.04	-	-	-	-	0.5±0.06	-	-	-
**3**	Sabinene	mh	975	-	-	0.1±0.01	-	0.3±0.03	-	0.6±0.18	-	-	-	-	-
**4**	β-Pinene	mh	979	-	-	-	-	0.7±0.05	-	-	-	-	-	-	-
**5**	1-Octen-3-ol	nt	980	-	0.3±0.04	1.2±0.02	0.5±0.04	-	1.6±0.22	-	-	-	-	-	-
**6**	3-Octanone	nt	984	-	-	0.2±0.02	-	-	-	-	-	-	-	-	-
**7**	6-Methyl-5-heptene-2-one	nt	986	-	-	-	0.4±0.03	-	-	-	-	-	-	-	-
**8**	β-Myrcene	mh	991	-	0.2±0.05	0.5±0.03	1.1±0.15	0.2±0.01	-	-	0.1±0.00	-	-	-	-
**9**	3-Octanol	nt	994	-	-	-	-	-	0.5±0.07	-	-	-	-	-	-
**10**	*o*-Cymene	mh	1026	-	-	-	-	-	0.2±0.04	-	-	0.2±0.15	-	-	-
**11**	Limonene	mh	1030	0.5±0.11	0.5±0.08	8.2±0.38	0.4±0.04	-	-	-	1.0±0.54	0.2±0.16	-	-	-
**12**	Eucalyptol	om	1031	-	-	-	-	10.0±0.68	0.5±0.05	-	0.2±0.15	1.4±0.14	0.1±0.01	-	1.4±0.43
**13**	*(E)*- β-Ocimene	mh	1050	-	-	-	0.2±0.03	0.5±0.02	-	-	-	-	1.3±0.48	-	0.5±0.22

**14**	γ-Terpinene	mh	1060	-	-	-	-	-	0.1±0.07	-	0.1±0.07	0.6±0.05	0.2±0.18	-	-
**15**	*cis*-Sabinene hydrate	om	1070	-	-	-	-	0.2±0.03	0.3±0.01	-	-	-	0.5±0.16	-	0.1±0.08
**16**	*cis*-linalool oxide (furanoid)	om	1087	-	-	-	-	-	0.1±0.03	-	-	-	-	-	-
**17**	Terpinolene	mh	1089	-	-	-	-	-	-	-	0.1±0.01	-	0.1±0.03	-	-
**18**	*p*-Cymenene	mh	1091	-	-	0.1±0.03	-	-	-	-	-	-	-	-	-
**19**	Linalool	om	1099	-	-	-	3.0±0.06	0.2±0.02	39.9±1.38	0.8±0.21	-	-	6.2±1.88	2.0±0.03	-
**20**	2-Methylbutyl 2-methylbutyrate	nt	1105	-	-	-	-	-	-	-	0.1±0.08	-	-	-	-
**21**	1-Octen-3-yl-acetate	nt	1113	-	-	0.5±0.03	-	-	-	-	-	-	-	-	-
**22**	*trans*-*p*-Mentha-2,8-diene-1-ol	om	1123	-	-	0.2±0.03	-	-	-	-	-	-	-	-	-
**23**	3-Octanol, acetate	nt	1124	-	-	-	-	-	-	1.2±0.29	-	-	-	-	-
**24**	Camphor	om	1145	-	-	-	0.3±0.02	-	-	-	-	4.2±0.10	0.6±0.18	-	-
**25**	*trans*-Verbenol	om	1146	-	-	0.1±0.09	-	-	-	-	-	-	-	-	-
**26**	*trans*-Chrysanthemal	nt	1153	-	-	-	0.5±0.01	-	-	-	-	-	-	-	-
**27**	Citronellal	om	1154	-	-	-	1.8±0.02	-	-	-	-	-	-	-	-
**28**	*p*-Menthone	om	1155	0.2±0.09	7.8±0.32	6.9±0.29	-	-	-	-	-	-	-	-	-
**29**	*iso*Menthone	om	1163	1.9±0.39	3.0±0.11	31.6±0.15	-	-	-	-	-	-	-	-	-
**30**	endo-Borneol	om	1167	-	-	-	0.8±0.03	-	0.1±0.01	-	-	-	-	-	-
**31**	*iso*Pulegone	om	1175	-	-	0.8±0.05	1.3±0.16	-	-	-	-	-	-	-	-
**32**	Terpinen-4-ol	om	1177	-	-	-	-	-	0.1±0.01	-	-	-	3.1±0.86	-	-
**33**	*iso*Geranial	om	1185	-	-	-	0.3±0.01	-	-	-	-	-	-	-	-
**34**	α-Terpineol	om	1189	-	-	-	0.1±0.02	0.3±0.02	1.3±0.07	-	0.1±0.09	-	0.2±0.00	0.7±0.03	0.3±0.10
**35**	Myrtenol	om	1195	-	-	-	-	-	-	-	0.1±0.07	-	-	-	-
**36**	Estragole	pp	1196	-	-	-	-	-	-	-	-	-	-	-	7.8±1.72
**37**	Verbenone	om	1205	-	0.5±0.02	-	-	-	-	-	-	-	-	-	-
**38**	*n*-Octyl acetate	nt	1214	-	-	-	-	-	-	-	-	-	0.2±0.17	-	-
**39**	β-Cyclocitral	ac	1220	-	-	-	-	-	-	-	0.1±0.08	-	-	-	-

**40**	8,9-Dehydrothymol	om	1221	-	0.3±0.04	-	-	-	-	-	-	-	-	-	-
**41**	Citronellol	om	1226	-	-	-	11.9±0.79	-	-	-	-	-	-	-	-
**42**	Thymol methyl ether	om	1235	-	-	-	-	-	17.7±0.20	-	-	-	-	-	-
**43**	Pulegone	om	1237	-	77.9±0.88	33.8±0.13	-	-	0.1±0.06	-	-	-	-	-	-
**44**	Neral	om	1240	-	-	-	-	-	-	-	-	-	-	38.3±0.89	-
**45**	β-Citral	om	1240	-	-	-	12.0±1.06	-	-	-	-	-	-	-	-
**46**	Carvacrol methyl ether	om	1245	-	-	-	-	-	0.2±0.03	-	-	-	-	-	-
**47**	Piperitone	om	1253	-	0.3±0.03	0.6±0.04	-	-	-	-	-	-	-	-	-
**48**	Geraniol	om	1255	-	-	-	10.6±0.54	-	-	-	-	-	-	-	-
**49**	Geranial	om	1270	-	-	-	-	-	-	-	-	-	-	44.2±1.87	-
**50**	Perilla aldehyde	om	1272	-	-	-	-	-	-	-	0.2±0.04	-	-	-	-
**51**	Citral	ac	1276	-	-	-	16.6±0.49	-	-	-	-	-	-	-	-
**52**	Methyl dihydrocinnamate	nt	1279	-	-	-	-	0.7±0.04	-	-	-	-	-	-	-
**53**	Bornyl acetate	om	1285	-	-	-	0.7±0.03	-	0.6±0.41	-	-	0.4±0.02	1.5±0.34	-	-
**54**	*cis,cis*-Nepetalactone	om	1289	-	-	-	-	9,1±0.21	-	-	-	-	-	-	-
**55**	Thymol	om	1290	-	-	-	-	-	24.7±1.14	-	-	-	-	-	-
**56**	*trans*-Sabinyl acetate	om	1291	-	-	0.2±0.03	-	-	-	-	-	-	-	-	-
**57**	*p*-Mentha-1,8-dien-7-ol	om	1297	-	-	-	-	-	-	-	0.5±0.05	-	-	-	-
**58**	Carvacrol	om	1299	-	-	-	-	-	2.8±0.22	-	-	-	-	-	-
**59**	Myrtenyl acetate	om	1327	-	-	-	-	-	-	-	1.4±0.42	-	0ò1±0.01	-	-
**60**	(-)-Dihydrocarvyl acetate	om	1330	-	-	-	0.3±0.03	-	-	-	-	-	-	-	-
**61**	*trans*-Carvyl acetate	om	1342	-	-	0.2±0.02	-	-	-	-	-	-	-	-	-
**62**	Piperitenone	om	1343	-	1.0±0.07	0.7±0.06	-	-	-	-	-	-	-	-	-
**63**	Ethyl dihydrocinnamate	nt	1353	-	-	-	-	0.2±0.03	-	-	-	-	-	-	-
**64**	Citronellyl acetate	om	1354	-	-	-	6.1±0.09	-	-	-	-	-	-	-	-
**65**	Eugenol	pp	1359	-	-	-	-	-	-	-	-	-	9.5±0.99	-	29.9±1.70

**66**	Neryl acetate	om	1362	-	-	-	3.1±0.10	-	-	-	-	-	-	-	-
**67**	α-Copaene	sh	1377	-	-	-	-	-	-	0.5±0.05	0.1±0.04	1.9±0.04	0.2±0.17	-	-
**68**	Geranyl acetate	om	1382	-	-	-	24.8±0.08	-	-	-	0.3±0.04	-	-	-	-
**69**	β-Bourbonene	sh	1388	0.4±0.02	-	-	-	0.2±0.02	-	-	-	-	-	-	-
**70**	β-Cubebene	sh	1389	-	-	-	-	-	-	-	-	-	0.2±0.18	-	-
**71**	β-Elemene	sh	1391	-	-	-	-	-	-	-	-	-	2.3±0.25	-	-
**72**	*cis*-Jasmone	nt	1393	-	-	0.1±0.02	-	-	-	-	-	-	-	-	-
**73**	Methyl perillate	om	1394	-	-	-	-	-	-	-	17.4±1.42	-	-	-	-
**74**	Methyleugenol	om	1402	-	-	-	0.5±0.04	-	-	-	-	-	0.5±0.06	-	-
**75**	β-Caryophyllene	sh	1419	7.6±0.43	5.5±0.29	1.8±0.07	1.3±0.07	12.4±0.05	0.5±0.05	30.9±0.81	11.2±0.97	5.7±0.07	0.5±0.04	1.3±0.22	2.7±0.13
**76**	α-Bergamotene	sh	1435	-	-	-	-	-	-	-	-	-	1.1±0.06	-	1.5±0.07
**77**	*p*-Mentha-1,8-dien-7-yl acetate	om	1436	-	-	-	-	-	-	-	25.3±1.54	-	-	-	-
**78**	α-Guaiene	sh	1440	-	-	-	-	-	-	-	-	-	2.0±0.07	-	-
**79**	α-Humulene	sh	1455	2.1±0.02	1.0±0.08	-	0.1±0.04	0.9±0.04	-	4.9±0.09	0.6±0.05	0.7±0.04	1.7±0.04	-	5.1±0.03
**80**	*(E)*-β-Famesene	sh	1457	-	1.1±0.09	-	-	2.8±0.10	-	0.3±0.02	-	-	0.9±0.05	-	0.8±0.05
**81**	*cis*-Muurola-4(15),5-diene	sh	1467	-	-	-	-	-	-	-	-	-	1.5±0.02	-	-
**82**	β-Cadinene	sh	1472	-	-	0.2±0.03	-	-	-	-	-	-	-	-	-
**83**	γ-Muurolene	sh	1477	-	-	-	-	-	-	-	-	0.8±0.04	-	-	-
**84**	γ-Curcumene	sh	1480	-	-	-	-	-	-	-	0.1±0.01	-	-	-	-
**85**	Germacrene D	sh	1485	33.9±0.95	2.0±0.14	2.5±0.06	-	8.8±0.08	4.6±0.41	19.0±0.03	-	-	14.6±0.69	-	4.7±0.06
**86**	phenylethyl 2-methylbutyrate	nt	1488	-	-	-	-	-	-	-	0.1±0.02	-	-	-	-
**87**	bicycloSesquiphellandrene	sh	1489	-	-	-	-	-	-	-	-	-	-	0.7±0.07	-
**88**	Ledene	sh	1493	-	-	-	-	-	-	-	0.5±0.02	-	-	-	-
**89**	α-Zingiberene	sh	1494	-	-	-	-	1.8±0.05	-	-	-	-	-	-	-
**90**	Aciphyllene	sh	1499	-	-	-	-	-	-	-	-	-	0.5±0.03	-	-
**91**	bicycloGermacrene	sh	1500	9.3±0.19	-	4.4±0.03	-	-	-	2.6±0.07	-	-	0.6±0.02	-	-

**92**	α-Muurolene	sh	1500	-	-	-	-	-	-	-	-	0.2±0.15	-	-	-
**93**	*iso*Daucene	sh	1503	-	-	-	-	0.4±0.02	-	-	-	-	-	-	0.2±0.03
**94**	*(E,E)*-α-Farnesene	sh	1506	0.9±0.09	-	-	-	-	-	-	-	-	-	-	-
**95**	β-Bisabolene	sh	1507	-	-	-	-	1.0±0.04	-	3.5±0.02	0.1±0.01	-	-	-	18.7±1.24
**96**	α-Farnesene	sh	1508	-	-	0.2±0.03	-	-	0.1±0.02	-	-	-	-	-	-
**97**	α-Bulnesene	sh	1510	1.5±0.00	-	-	-	-	-	1.4±0.00	-	-	10.0±0.44	-	-
**98**	*trans*-α-Bisabolene	sh	1512	-	-	-	-	-	-	-	-	-	-	1.3±0.14	-
**99**	γ-Cadinene	sh	1514	0.2±0.08	-	-	-	0.2±0.03	-	0.5±0.02	0.7±0.02	1.1±0.04	7.1±0.45	-	-
**100**	β-Sesquiphellandrene	sh	1523	-	-	-	-	0.9±0.04	-	0.9±0.01	-	-	-	-	0.2±0.01
**101**	δ-Cadinene	sh	1524	2.3±0.12	-	-	-	-	-	-	1.1±0.03	2.8±0.07	0.3±0.03	-	-
**102**	*trans*-γ-Bisabolene	sh	1531	-	-	-	-	0.2±0.03	-	-	-	-	-	-	-
**103**	α-Cadinene	sh	1538	-	-	-	-	-	-	-	-	-	0.1±0.01	-	-
**104**	Elemol	os	1550	-	-	-	-	-	-	0.6±0.06	-	-	-	-	-
**105**	myrtenyl 2-methyl butyrate	nt	1560	-	-	-	-	-	-	-	0.3±0.01	-	-	-	-
**106**	Germacrene B	sh	1562	-	-	-	-	-	-	-	-	-	-	-	22.1±2.26
**107**	*(E)*-Nerolidol	os	1564	-	-	-	-	-	0.2±0.03	-	0.2±0.01	-	1.2±0.06	-	-
**108**	Germacrene D-4-ol	os	1576	29.1±0.81	0.5±0.06	3.3±0.01	-	0.2±0.02	-	-	-	-	0.4±0.03	-	-
**109**	Caryophyllene oxide	os	1583	-	-	-	-	2.8±0.15	-	1.2±0.04	0.7±0.01	0.5±0.03	-	-	-
**110**	Viridiflorol	os	1591	-	-	-	-	-	0.1±0.06	-	-	0.4±0.02	-	-	-
**111**	*iso*Aromadendrene epoxide	os	1594	-	-	0.1±0.01	-	-	-	-	-	-	-	-	-
**112**	Guaiol	os	1597	-	-	-	-	-	-	-	-	28.9±0.04	-	-	-
**113**	Zingiberenol	os	1616	-	-	-	-	1.3±0.06	-	-	-	-	-	-	-
**114**	Juneol	os	1617	-	-	-	-	-	-	-	0.3±0.02	-	-	-	-
**115**	1,10-Di-*epi*-Cubenol	os	1619	-	-	-	-	-	-	-	-	-	3.2±0.33	-	-
**116**	Valencene	sh	1624	-	-	-	-	-	-	-	-	0.8±0.03	-	-	-
**117**	10-*epi*-γ-Eudesmol	os	1631	-	-	-	-	-	-	-	-	2.9±0.89	-	-	-

**128**	Selin-6-en-4alpha-ol	os	1636	-	-	-	-	-	-	-	1.6±0.05	-	-	-	-
**119**	Hinesol	os	1638	-	-	-	-	-	-	-	-	6.7±3.03	-	-	-
**120**	T-cadinol	os	1640	-	-	-	-	1.3±0.01	-	-	-	-	22.4±3.64	-	-
**121**	T-Muurolol	os	1642	2.6±0.42	-	0.1±0.02	-	-	-	-	-	-	-	-	-
**122**	δ-Cadinol	os	1643	0.2±0.12	-	-	-	-	-	-	-	-	-	-	-
**123**	1,3a-ethanol(1H)indien-4-ol, otahydro-2,2,4,7a-tetramethyl	os	1648	-	-	-	-	-	-	-	0.2±0.01	-	-	-	-
**124**	β-Eudesmol	os	1652	-	-	-	-	-	-	-	-	15.5±1.36	0.8±0.18	-	-
**125**	α-Eudesmol	os	1652	-	-	-	-	-	-	-	-	11.8±1.64	-	-	-
**126**	α-Cadinol	os	1654	6.8±1.03	-	0.3±0.01	-	-	-	-	-	-	1.0±0.19	-	-
**127**	Agarospirol	os	1655	-	-	-	-	-	-	0.5±0.02	-	1.9±0.06	-	-	-
**128**	Bulnesol	os	1672	-	-	-	-	-	-	1.2±0.09	-	-	-	-	-
**129**	Aromadendrene oxide-(2)	os	1678	-	-	-	-	0.6±0.04	-	-	-	-	-	-	-
**130**	Elemol acetate	os	1681	-	-	-	-	-	-	24.3±2.56	-	-	-	-	-
**131**	(+)-Valeranone	os	1684	-	-	-	-	-	-	-	-	1.9±0.02	-	-	-
**132**	α-Bisabolol	os	1686	-	-	-	-	-	-	-	0.4±0.02	-	0.5±0.09	-	1.5±0.20
**133**	ent-Germacra-4(15),5,10(14)-trien-1β-ol	os	1695	-	-	-	-	-	0.3±0.05	-	-	-	-	-	-
**134**	Shybunol	os	1701	-	-	-	-	-	-	-	3.3±0.17	-	-	-	-
**135**	β-Sinensal	os	1712	-	-	-	-	9.0±0.13	-	-	-	-	-	-	-
**136**	*(Z)*-α-*trans*-Bergamotol	os	1721	-	-	-	-	14.1±0.24	-	-	-	-	-	-	-
**137**	*(Z)*-Nuciferol	os	1735	-	-	-	-	0.9±0.03	-	-	-	-	-	-	-
**138**	*(Z)*-β-Curcumen-12-ol	os	1746	-	-	-	-	6.3±0.01	-	-	-	-	-	-	-
**139**	(6R,7R)-Bisabolone	os	1747	-	-	-	-	0.4±0.02	-	-	-	-	-	-	-
**140**	*cis*-Lanceol	os	1763	-	-	-	-	4.1±0.06	-	-	-	-	-	-	-
**141**	β-Costol	os	1778	-	-	-	-	3.3±0.05	-	-	-	-	-	-	-
**142**	Eugenyl isovalerate	pp	1864	-	-	-	-	-	-	-	-	-	-	-	0.1±0.07
**143**	*iso*Pimara-9(11),15-diene	dh	1906	-	-	-	-	-	-	-	1.0±0.02	-	-	-	-

**144**	pentylcurcumene	dh	1910	-	-	-	-	-	-	-	5.9±0.69	-	-	-	-
**145**	Farnesyl acetone	ac	1920	-	-	-	-	-	-	-	0.5±0.06	-	-	-	-
**146**	Phytol	od	1943	-	0.1±0.01	0.3±0.03	-	0.5±0.05	1.2±0.19	-	-	-	0.1±0.05	-	0.2±0.04
**147**	Abietatriene	dh	2053	-	-	-	-	-	-	-	0,3±0.06	-	-	-	-
**148**	5α-Androst-16-en-3-one	od	2160	-	-	-	-	-	-	-	0.5±0.04	-	-	-	-
**149**	Podocarpa-8,11,13-triene-7β,13-diol, 14-isopropyl-	nt	2324	-	-	-	-	-	-	-	1.0±0.22	-	-	-	-
**150**	Ferruginol	od	2330	-	-	-	-	-	-	-	6.7±1.34	0.5±0.04	-	-	-
**151**	Abietol	os	2381	-	-	-	-	-	-	-	-	0.2±0.16	-	-	-
**152**	Tetracosane	nt	2400	-	-	-	-	-	0.3±0.05	1.5±0.50	0.3±0.07	0.2±0.16	-	11.5±0.54	0.1±0.08
**153**	Pentacosane	nt	2500	-	-	-	-	-	0.4±0.03	-	-	1.7±0.04	-	-	-
				**Mentheae Tribe**									**Ocimeae Tribe**		
				**Subtribe Nepetinae**					**Subtribe Menthinae**	**Subtribe Salviinae**			***Subtribe Ociminae***		
				***A.* ‘Arcado Pink’**	***A. aurantiaca***	***A.* ‘BleuBoa’**	***A. mexicana***	***N. x faasanii***	***M. didyma***	***S. discolor***	***S. dorisiana***	***S. microphylla***	***O. b* ‘Cinnamon’**	***O. × citriodorum***	***O. b.* ‘Blue Spice’**
**Monoterpene Hydrocarbons (mh)**				0.5±0.11	0.7±0.13	8.9±0.39	2.0±0.25	1.9±0.10	0.3±0.10	0.6±0.21	1.3±0.82	1.9±0.47	1.6±0.78	2.0±0.03	0.5±0.22
**Oxygenated Monoterpenes (om)**				2.1±0.57	**88.8±0.35**	**75.1±0.28**	**77.6±0.67**	19.8±0.43	**88.4±0.28**	0.8±0.21	45.5±2.81	6.0±0.21	12.3±3.85	**83.2±0.98**	1.8±0.60
**Sesquiterpene hydrocarbons (sh)**				**58.1±1.23**	9.6±0.59	9.1±0.23	1.4±0.21	29.6±0.43	5.2±0.46	**64.5±0.86**	14.4±1.10	14.0±0.46	43.6±1.15	3.3±0.42	**56.0±3.32**
**Oxygenated Sesquiterpenes (os)**				38.7±2.47	0.5±0.06	3.8±0.02	-	44.3±0.10	0.6±0.13	27.8±2.73	6.7±0.26	**75.8±0.20**	29.6±4.65	-	1.5±0.20
**Diterpene Hydrocarbons (dh)**				-	-	-	-	-	-	-	7.2±0.74	-	-	-	-
**Oxygenated Diterpenes (od)**				-	0.1±0.01	0.3±0.03	-	0.5±0.05	1.2±0.19	-	7.2±1.81	-	-	-	0.2±0.04
**Non-terpene derivatives (nt)**				-	0.3±0.04	2.0±0.08	1.4±0.07	0.9±0.07	2.8±0.64	2.7±0.79	1.8±0.08	1.9±0.14	0.2±0.17	11.5±0.54	0.1±0.08
**Phenylpropanoids (pp)**				-	-	-	-	-	-	-	-	-	10.0±1.05	-	37.8±3.36
**Apocarotenoids (ac)**				-	-	-	16.6±0.49	-	-	-	0.6±0.02	-	-	-	-
**Total Identified**				**99.4±0.56**	**100.0±0.0**	**99.2±0.26**	**99.0±0.11**	**97.0±0.09**	**98.5±0.24**	**96.4±0.30**	**84.7±2.80**	**99.8±0.21**	**97.3±0.04**	**100.0±0.0**	**97.9±0.70**

[1]Compounds present in tables with value > 1% in at least one of studied species; [2]LRI: relative retention index determined on HP-5MS capillary column.
